# Bibliographical

**Published:** 1858-05

**Authors:** 


### Bibliographical.

We have received "Outlines of a course of Lectures on the Principles and Practice of Surgery," delivered by E. Geddings, M. D., Prof. of Surgery in the Medical College of South Carolina. Prepared by Thos. S. Waring, M. D., and Samuel Logan, M. D., from notes taken during the course. Published with the consent of, and revised by, Prof. Geddings. Charleston: S. G. Courtenay & Co. The mere title, "Geddings' Lectures on Surgery," would be a sufficient recommendation of this work, as there is no one more favorably known to the profession, particularly in the South, for sterling worth and practical good sense than Prof. E. Geddings, of Charleston. It is a most excellent publication, and reflects great credit upon the young gentlemen, Drs. Waring and Logan. It abounds in practical truths, to the exclusion of contested theories—a feature which should highly recommend it to students and young practitioners. To those who have attended, or expect to attend a course of lectures in the South Carolina Medical College, it is invaluable.

The mechanical execution is highly creditable to the publishers, Messrs. S. G. Courtenay & Co., Charleston, South Carolina.

We have received from the publishers, Messrs. Blanchard & Lea, of Philadelphia, Plates illustrative of Wilson on "Diseases of the Skin; 4th edition."

This work is most admirably and beautifully gotten up, and contains 19 plates, embracing the "Structure of the Scarf Skin," "Anatomy of the Sensitive Skin and Nail," "Anatomy of the Sebiparous Glands," "Anatomy of the Hair," "The Acarus Scabiei, or Itch Animalcule," "Structure of Warts and Corns, together with some diseases of the Sebiparous Glands," "Congestive Inflammation of the Derma, comprising Urticaria, Rubeola, and Erythema," "Effusive Inflammation of the Derma, comprising Pemphigus and Rupia, Herpes and Eczema," "Suppurative Inflammation of the Derma, Impetigo and Ecthyma," "Depositive Inflammation of the Derma, Lichen, Strophulus and Prurigo," "Squamous Inflammation of the Derma—Lepra, Psoriasis, Pityriasis," "Lupus non Exedens," "Diseases of the Hair-Follicles and Hairs—Acne, Sycosis, Favus, Trichosis," "Ex-anthematous and Papular Syphilitic Eruptions."

This, as well as the large text, which it is intended to accompany, we would commend to the profession, especially to the junior practitioner, who is so often at a loss to determine the true character of skin diseases.

In addition to the above, we have also received from the same publishers, Messrs. Blanchard & Lea, Philadelphia, too late for notice in the present number, a new and enlarged edition of "Dunglisons Medical Dictionary," "A Manual of Medical Diagnosis, being an analysis of the Signs and Symptoms of Disease, by A. W. Barclay, M. D., &c.;" also, "Miller's System of Obstetrics." All of which will be noticed at length, so soon as we shall be able to give them the proper attention.

We have also received "*Life;* its relations, animal and mental, an inaugural Dissertation by J. Dickson Bruns, A.M., M.D., Charleston, S. C.; and an *Essay* on prolapse of the Funis, with a new method of treatment, by T. Gaillard Thomas, M. D., Physician to the Demilt Dispensary, New York City, read February 3d, 1858, before the New York Academy of Medicine.

